# Reviewed article: Teixeira MMP, Santos DF, Barros EA, Santos LH, Siqueira LS, Pascoal LM, Costa ACPJ, Neto MS. Factors associated with deaths due to visceral leishmaniasis among children at a hospital in the southwest of Maranhão state: a cross-sectional study, Imperatriz, 2010-2021. Epidemiol Serv Saude. 2025:34;e20240738

**DOI:** 10.1590/S2237-96222025v34e20240738.b

**Published:** 2025-09-29

**Authors:** Sílvia Leticia Cerqueira de Jesus

**Affiliations:** 1 Secretaria Municipal da Saude de Salvador, Salvador, BA, Brazil Secretaria Municipal da Saude de Salvador Salvador BA Brazil

## Peer review B

## Questionnaire

Does the manuscript contain new and significant information to justify publication? Yes

Does the Abstract (Summary) clearly and accurately describe the content of the article? Yes

Is the problem significant and concisely stated? Yes

Are the methods described comprehensively? Yes

Are the interpretations and conclusions justified by the results? Yes

Is adequate reference made to other work in the field? Yes

## Manuscript Structure

Length of article is: Adequate

Number of tables is: Adequate

Number of figures is: Adequate

Please state any conflict(s) of interest that you have in relation to the review of this paper. None

Interest: Excellent

Quality: Excellent

Originality: Excellent

Overall: Excellent

Recommendation: Major Revision

## Comments

O texto está bom, mas tem necessidade de pequenos ajustes. É necessário reescrever o texto da metodologia, que versa sobre a busca dos dados, pois ficou confuso, tanto no resumo, quanto no manuscrito. é necessário deixar claro que foram coletados no SINAN ,óbitos por LV, na faixa etária de 0-12 anos , em um hospital do sudoeste maranhense.

Em relação aos óbitos, é dito na metodologia que houveram 48 óbitos e , na discursão , apareceram somente 43. Portanto esta faltando 5 óbitos. Isso precisa ser explicado.

Em relação à faixa etária ( idade acometida ), o texto fala que o estudo foi realizado em faixa etária menor que 12 anos , mas quando é olhado na tabela , os autores analisaram apenas 0-4 anos . Só foi achado óbito de 0-4 anos? ou não foi estudado pessoas de 0-12 anos? Ficou confuso. Outra sugestão, é destrinchar os óbitos por faixa etária, e dizer qual foi a faixa etária que mais veio a óbito, neste período, nesse hospital. Justamente para corroborar com os dados apresentados nesse estudo, que evidencia que faixa etária de 1-4 anos é fator de proteção. Na faixa etária de 0-12 anos, qual foi a idade mais acometida? Como no SINAN não existe faixa etária de 0-12, sugiro que destrinche por idade.

Outros artigos que versam sobre óbitos por LV, trazem vômito, tosse e diarreia como fator de gravidade e, portanto, diretamente associados ao óbito e que não são variáveis disponíveis no SINAN. Sendo assim, como vocês fizeram estudo em um hospital e usaram somente dados do SINAN, seria interessante avaliar portuário, para ver quais foram os outros fatores que esses pacientes tiveram (se teve vômito, tosse, diarreia etc).

Em relação a referência, sentir falta de referenciar José Ângelo Lauletta Lindoso, da professora Dorcas Lamounier Costa, que são referência nacional para LVH.

